# Emergency Dispatches for Suicide Attempts During the COVID-19 Outbreak in Okayama, Japan: A Descriptive Epidemiological Study

**DOI:** 10.2188/jea.JE20210066

**Published:** 2021-09-05

**Authors:** Hiroshi Habu, Soshi Takao, Ryohei Fujimoto, Hiromichi Naito, Atsunori Nakao, Takashi Yorifuji

**Affiliations:** 1 Department of Epidemiology, Okayama University Graduate School of Medicine, Dentistry and Pharmaceutical Sciences, Okayama, Japan; 2 Department of Cardiovascular Medicine, Okayama University Hospital, Okayama, Japan; 3 Department of Emergency and Critical Care and Disaster Medicine, Okayama University Graduate School of Medicine, Dentistry and Pharmaceutical Sciences, Okayama, Japan

**Keywords:** COVID-19, epidemiology, emergency medical dispatch, suicide

## Abstract

**Background:**

Hardships associated with the ongoing coronavirus disease 2019 (COVID-19) pandemic can affect mental health, potentially leading to increased risk of suicide. We examined the relationship between the COVID-19 outbreak and suicide attempts in Okayama, Japan using information from emergency dispatches.

**Methods:**

This was a descriptive epidemiological study. We collected information on emergency dispatches in Okayama City and Kibichuo from March to August in 2018, 2019, and 2020 (*n* = 47,770 cases). We compared emergency dispatches and their demographic characteristics, especially focusing on suicide attempts, during these 3 years.

**Results:**

The number of emergency dispatches in 2020 decreased compared with the previous 2 years, while the number and proportion of emergency dispatches related to suicide attempts increased. This increase was more pronounced among women and those aged 25–49 years. Among women aged 25–49 years, there was a cumulative total of 43 suicide attempts in 2018 and 2019 and 73 suicide attempts in 2020.

**Conclusions:**

The number and proportion of emergency dispatches related to suicide attempts increased in 2020 compared with the previous 2 years, especially among women and those aged 25–49 years. This increase may be partly explained by hardships, such as economic losses or reduced social ties, during the COVID-19 outbreak.

## INTRODUCTION

Coronavirus disease 2019 (COVID-19) is now a global pandemic. Although the impact in Japan seems to be minimal (53 deaths per 1 million individuals as of February 9, 2021, substantially lower than in other countries),^1^^,^^2^ Japanese society has borne hardships, such as reduced opportunity for education, decreased social interactions, and diminished gross domestic product because of several regulations against COVID-19.^3^^,^^4^ It has been speculated that these hardships can affect the mental health of residents,^5^^–^^7^ potentially leading to increased risk of suicidal ideation, suicide attempts, or suicide.^8^^–^^12^

Several studies have already reported increased risks of suicide or suicidal ideation during the COVID-19 pandemic in Western and Asian countries, including Japan.^13^^–^^17^ However, fewer studies have evaluated associations between the COVID-19 pandemic and suicide attempts, which may be a more specific marker of mental hardship within the general population. One study conducted in Ireland evaluated a 3-month change in emergency department presentations for self-harm from March to May, 2020, and documented an initial reduction followed by an increase in May.^18^ Thus, a longer observation period may be needed to understand the impact of the COVID-19 pandemic on suicide attempts.

In the present study, we examined the relationship between the COVID-19 outbreak and suicide attempts over a 6-month period (March to August) in 2018, 2019, and 2020. We used information from emergency dispatches to evaluate the effects of the outbreak on suicide attempts over this period.

## METHODS

### Study design and subjects

This was a descriptive epidemiological study. The Emergency Section of the Fire Bureau in Okayama City is in charge of Okayama City and Kibichuo (total population in 2018–2020 was about 720,000), provided electronic data (stripped of patient names) on all ambulance calls (including the cases of already dead on arrival) from January 2018 until August 2020. We carried out analysis of emergencies in Okayama City or Kibichuo according to data from March 1 until August 31 in 2018, 2019, and 2020. Overall, 48,649 cases were analyzed. Okayama City and Kibichuo are in the southern part of Okayama Prefecture, which is located in western Japan and has a population of about 1.9 million.

### Data collection

For each case, the data included the time of the emergency call, the location of occurrence, sex, age, incident type, injury form, and degree of severity (mild, moderate, severe, death, or other). Because the exact time of onset was unavailable, we used the time of the emergency call as the event onset for each case. Incident type was the reason for the emergency call and included acute disease, general injury, traffic accidents, fires, suicide attempts, and homicides. Injury form was the type of injury (eg, car accidents, gas poisoning, or chemicals). The emergency medical technicians on the ambulance crew recorded and corrected all information as necessary. We excluded cases with missing information on sex or age, leaving 47,770 cases for the analysis.

### Descriptive statistics

We first described demographic characteristics of the study subjects in each of the 3 years. We categorized age into 5 categories (≤14, 15–24, 25–49, 50–64, and ≥65 years). First, we divided the participants into three categories (young: ≤14, working-age: 15–64, and elderly: ≥65 years), which are often used as age categories in Japan.^19^ Then, we further focused on the working-age population to examine the impact of the pandemic on that particular generation.^20^ We also evaluated the numbers and proportions of incident types of suicide attempts in each study year. We described the monthly number of emergency dispatches for all causes and for suicide attempts in each year.

We next restricted our analysis to cases who attempted suicide and described their demographic characteristics during the 3 study years. For this analysis, we calculated percent incremental increase in 2020 compared with the previous 2 years using the following formula: (number in 2020/((number in 2018 + number in 2019)/2)) * 100 − 100. We next evaluated the (relative) frequencies of injury form (chemicals; firearms, knives, sharp objects, and blunt instruments; hanging; jumping; drowning; gas; or other) and degree of severity (death or other). The category of “chemicals” included both drug overdose and chemical poisoning. We also examined the (relative) frequencies of injury form and degree of severity by age and sex.

Stata SE version 16.1 (StataCorp, College Station, TX, USA) was used for all calculations. The study was approved by the Institutional Review Board of Okayama University Graduate School of Medicine, Dentistry, and Pharmaceutical Sciences (No. 2010-025).

## RESULTS

**Figure 1.**
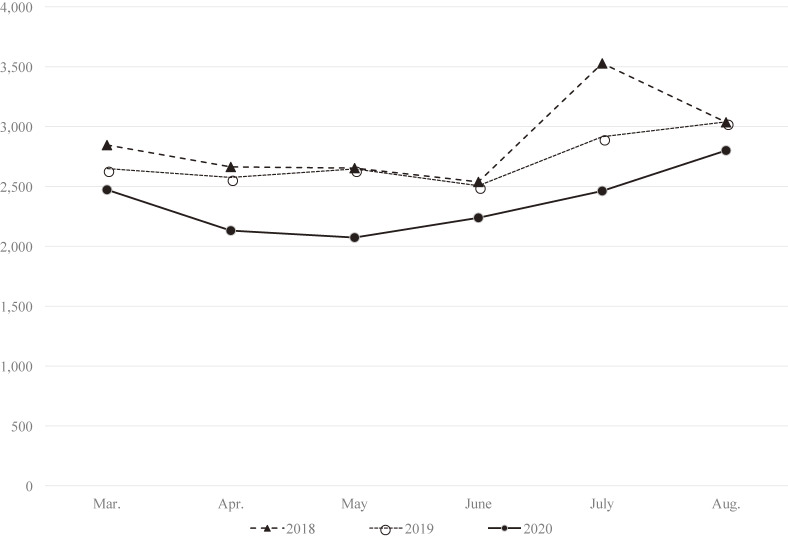
Monthly number of emergency dispatches in Okayama City and Kibichuo from March to August in 2018, 2019, and 2020

**Figure 2.**
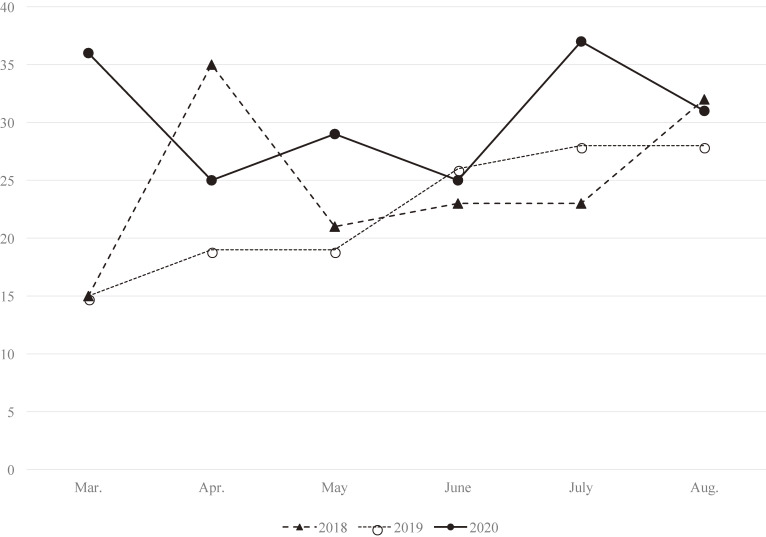
Monthly number of emergency dispatches related to suicide attempts in Okayama City and Kibichuo from March to August in 2018, 2019, and 2020

The number of emergency dispatches decreased in 2020 compared with the numbers in 2018 and 2019 (Table 1). This was true for both sexes and all age categories. The decrease was most prominent among individuals under 25 years of age. However, the number and proportion of emergency dispatches related to suicide attempts increased in 2020 (1.3%) compared with 2018 (0.9%) and 2019 (0.8%). Although there was no marked difference in all-cause monthly emergency dispatches in each year (Figure 1), the number of dispatches for suicide attempts increased in March and July 2020 compared with the previous 2 years (Figure 2).

**Table 1.** Demographic characteristics of study subjects obtained from information on emergency dispatches in Okayama City and Kibichuo, March–August 2018, 2019, and 2020 (*n* = 47,770)

	2018	2019	2020
	(*n* = 17,264)	(*n* = 16,330)	(*n* = 14,176)
	(*N*^a^ = 721,025)	(*N* = 720,772)	(*N* = 720,168)
Area, *n* (%)			
Okayama city	16,922 (98)	15,999 (98)	13,901 (98.1)
Kibichuo	342 (2)	331 (2)	275 (1.9)
Sex, *n* (%)			
Men	8,826 (51.1)	8,413 (51.5)	7,195 (50.8)
Women	8,438 (48.9)	7,917 (48.5)	6,981 (49.3)
Age, years, mean (SD)	59.6 (26.8)	60.6 (26.8)	62.8 (25.3)
Age, *n* (%)			
≤14	1,222 (7.1)	1,136 (7)	685 (4.8)
15–24	1,483 (8.6)	1,347 (8.3)	944 (6.7)
25–49	3,011 (17.4)	2,626 (16.1)	2,457 (17.3)
50–64	2,158 (12.5)	2,081 (12.7)	1,856 (13.1)
≥65	9,390 (54.4)	9,140 (56)	8,234 (58.1)
Incident type, *n* (%)			
Others	17,115 (99.1)	16,195 (99.2)	13,993 (98.7)
Suicide attempts	149 (0.9)	135 (0.8)	183 (1.3)

SD, standard deviation.

^a^*N* is the sum total population of Okayama City and Kibichuo residents. These calculations are based on the annual data as of January 1 of the Basic Resident Registration.

Restricting the analysis to suicide attempts (Table 2) showed that the number of dispatches increased in 2020 compared with the previous 2 years, especially among women and those aged 25–49 years. Among women aged 25–49 years, the number of suicide attempts increased from a cumulative total of 43 cases in both 2018 and 2019 to 73 cases in 2020. The incremental percentage increase in suicide attempts in 2020 was 38.5% among women (11.8% in men) and 71.0% among those aged 25 to 49 years. In terms of injury form, chemicals and instruments (firearms, knives, sharp objects, and blunt instruments) showed incremental increases of 32.1% and 45.5%, respectively. Despite the increase in the number of suicide attempts, the number of suicide deaths did not differ between years.

**Table 2.** Characteristics of subjects who attempted suicide obtained from information on emergency dispatches in Okayama City and Kibichuo, March–August 2018, 2019, and 2020 (*n* = 467)

	2018	2019	2020	% increment in 2020 vs 2018/2019^c^
	(*n* = 149)	(*n* = 135)	(*n* = 183)	
Sex, *n* (%)				
Men	54 (36.2)	48 (35.6)	57 (31.2)	11.8
Women	95 (63.8)	87 (64.4)	126 (68.9)	38.5
Age, *n* (%)				
≤14	2 (1.3)	1 (0.7)	2 (1.1)	33.3
15–24	27 (18.1)	31 (23)	29 (15.9)	0.0
25–49	67 (45)	57 (42.2)	106 (57.9)	71.0
50–64	35 (23.5)	24 (17.8)	21 (11.5)	▲ 28.8
≥65	18 (12.1)	22 (16.3)	25 (13.7)	25.0
Age in men, *n* (%)				
≤14	1 (1.9)	1 (2.1)	0 (0)	▲ 100.0
15–24	8 (14.8)	10 (20.8)	6 (10.5)	▲ 33.3
25–49	24 (44.4)	14 (29.2)	33 (57.9)	73.7
50–64	16 (29.6)	14 (29.2)	5 (8.8)	▲ 66.7
≥65	5 (9.3)	9 (18.8)	13 (22.8)	85.7
Age in women, *n* (%)				
≤14	1 (1.1)	0 (0)	2 (1.6)	300.0
15–24	19 (20)	21 (24.1)	23 (18.3)	15.0
25–49	43 (45.3)	43 (49.4)	73 (57.9)	69.8
50–64	19 (20)	10 (11.5)	16 (12.7)	10.3
≥65	13 (13.7)	13 (14.9)	12 (9.5)	▲ 7.7
Injury form, *n* (%)^a^				
Chemicals	62 (42.5)	50 (30)	74 (40.4)	32.1
Firearms, knives, sharp objects, blunt instruments	26 (17.8)	29 (21.5)	40 (21.9)	45.5
Hanging	34 (23.3)	30 (22.2)	36 (19.7)	12.5
Jumping	2 (1.4)	12 (8.9)	8 (4.4)	14.3
Drowning	1 (0.7)	3 (2.2)	5 (2.7)	150.0
Gas	1 (0.7)	1 (0.7)	4 (2.2)	300.0
Others	20 (13.7)	10 (14.4)	16 (8.7)	6.7
Severity, *n* (%)^b^				
Dead	13 (11.4)	13 (12.2)	13 (9)	0.0
Others	101 (88.6)	94 (87.9)	131 (91)	34.4

^a^There were missing data for 3 subjects in 2018.

^b^There were missing data for 35 subjects in 2018, 28 subjects in 2019, and 39 subjects in 2020.

^c^Incremental % increase was calculated using the following formula: (number in 2020/((number in 2018 + number in 2019)/2)) * 100 − 100.

Further analysis of the sex- and age-specific trends in suicide attempts showed that there was an increase in the use of instruments, such as knives and sharp objects, among men of all ages (4 cases in 2018, 3 cases in 2019, and 14 cases in 2020) (Table 3). Over the same period, there was an increase in chemical use in suicide attempts among women (Table 4).

**Table 3.** Characteristics of suicide attempts by severity among men obtained from information on emergency dispatches in Okayama City and Kibichuo, March–August 2018, 2019, and 2020 (*n* = 102)^a^

	2018		2019		2020	
	(*n* = 32)		(*n* = 33)		(*n* = 37)	
		
	Dead	Others	Dead	Others	Dead	Others
	(*n* = 6)	(*n* = 26)	(*n* = 7)	(*n* = 26)	(*n* = 9)	(*n* = 28)
Age, *n* (%)						
≤14	0 (0)	1 (3.85)	0 (0)	1 (0.85)	0 (0)	0 (0)
15–24	1 (16.67)	1 (3.85)	0 (0)	8 (30.77)	1 (11.11)	2 (7.14)
25–49	2 (33.33)	11 (42.31)	2 (28.57)	8 (30.77)	2 (22.22)	18 (64.29)
50–64	1 (16.67)	11 (42.31)	3 (42.86)	6 (23.08)	2 (22.22)	1 (3.57)
≥65	2 (33.33)	2 (7.69)	2 (28.57)	3 (11.54)	4 (44.44)	7 (25)
Injury form, *n* (%)						
Total	6 (100)	26 (100)	7 (100)	26 (100)	9 (100)	28 (100)
Chemicals	0 (0)	12 (46.15)	0 (0)	10 (38.46)	0 (0)	7 (25)
Firearms, knives, sharp objects, blunt instruments	0 (0)	4 (15.38)	0 (0)	3 (11.54)	1 (11.11)	14 (50.01)
Hanging	5 (83.33)	2 (7.69)	5 (71.43)	4 (15.38)	6 (66.67)	1 (3.57)
Jumping	1 (16.67)	1 (3.85)	1 (14.29)	5 (19.23)	2 (22.22)	2 (7.14)
Drowning	0 (0)	0 (0)	0 (0)	2 (7.69)	0 (0)	1 (3.57)
Gas	0 (0)	0 (0)	0 (0)	1 (3.85)	0 (0)	2 (7.14)
Others	0 (0)	7 (26.93)	1 (14.29)	1 (3.85)	0 (0)	1 (3.57)
Injury form, age 25–49, *n* (%)						
Total	2 (100)	11 (100)	2 (100)	8 (100)	2 (100)	18 (100)
Chemicals	0 (0)	3 (27.27)	0 (0)	3 (37.5)	0 (0)	5 (27.78)
Firearms, knives, sharp objects, blunt instruments	0 (0)	2 (18.18)	0 (0)	1 (12.5)	0 (0)	9 (50)
Hanging	1 (50)	1 (9.09)	1 (50)	2 (25)	1 (50)	0 (0)
Jumping	1 (50)	0 (0)	0 (0)	1 (12.5)	1 (50)	2 (11.11)
Drowning	0 (0)	0 (0)	0 (0)	1 (12.5)	0 (0)	0 (0)
Gas	0 (0)	0 (0)	0 (0)	0 (0)	0 (0)	2 (11.11)
Others	0 (0)	5 (45.45)	1 (50)	0 (0)	0 (0)	0 (0)

^a^There were missing data for 22 subjects in 2018, 15 subjects in 2019, and 20 subjects in 2020.

**Table 4.** Characteristics of suicide attempts by severity among women obtained from information on emergency dispatches in Okayama City and Kibichuo, March–August 2018, 2019, and 2020 (*n* = 263)^a^

	2018		2019		2020	
	(*n* = 82)		(*n* = 74)		(*n* = 107)	
		
	Dead	Others	Dead	Others	Dead	Others
	(*n* = 7)	(*n* = 75)	(*n* = 6)	(*n* = 68)	(*n* = 4)	(*n* = 103)
Age, *n* (%)						
≤14	1 (14.29)	0 (0)	0 (0)	0 (0)	1 (25)	1 (0.97)
15–24	0 (0)	15 (20)	2 (33.33)	16 (23.53)	0 (0)	21 (20.39)
25–49	3 (42.86)	32 (42.67)	4 (66.67)	36 (52.94)	1 (25)	61 (59.22)
50–64	1 (14.29)	18 (24)	0 (0)	9 (13.24)	0 (0)	11 (10.68)
≥65	2 (28.57)	10 (13.33)	0 (0)	7 (10.29)	2 (50)	9 (8.74)
Injury form, *n* (%)						
Total	7 (100)	75 (100)	6 (100)	68 (100)	4 (100)	103 (100)
Chemicals	0 (0)	45 (60)	0 (0)	37 (54.41)	0 (0)	64 (62.14)
Firearms, knives, sharp objects, blunt instruments	0 (0)	17 (22.67)	2 (33.33)	16 (23.53)	0 (0)	18 (17.48)
Hanging	6 (85.71)	3 (4)	2 (33.33)	5 (7.35)	4 (100)	5 (4.85)
Jumping	0 (0)	0 (0)	2 (33.33)	4 (5.88)	0 (0)	3 (2.91)
Drowning	0 (0)	1 (1.33)	0 (0)	1 (1.47)	0 (0)	3 (2.91)
Gas	1 (14.29)	0 (0)	0 (0)	0 (0)	0 (0)	0 (0)
Others	0 (0)	9 (12)	0 (0)	5 (7.36)	0 (0)	10 (9.71)
Injury form, age 25–49, *n* (%)						
Total	3 (100)	32 (100)	4 (100)	36 (100)	1 (100)	61 (100)
Chemicals	0 (0)	22 (68.75)	0 (0)	20 (50)	0 (0)	38 (67.21)
Firearms, knives, sharp objects, blunt instruments	0 (0)	5 (15.63)	2 (50)	8 (33.33)	0 (0)	14 (22.95)
Hanging	2 (66.67)	1 (3.13)	1 (25)	4 (5.56)	1 (100)	1 (1.64)
Jumping	0 (0)	0 (0)	1 (25)	1 (0)	0 (0)	0 (0)
Drowning	0 (0)	0 (0)	0 (0)	0 (0)	0 (0)	0 (0)
Gas	1 (33.33)	0 (0)	0 (0)	0 (0)	0 (0)	0 (0)
Others	0 (0)	4 (12.49)	0 (0)	3 (11.11)	0 (0)	5 (8.2)

^a^There were missing data for 13 subjects in 2018, 13 subjects in 2019, and 19 subjects in 2020.

## DISCUSSION

In the present study, we examined the relationship between the COVID-19 outbreak and suicide attempts in Okayama, Japan over a 6-month period (March to August) in 2018, 2019, and 2020 using information from emergency dispatches. We found that the number of emergency dispatches in 2020 decreased compared with the previous 2 years, while the number and the proportion of emergency dispatches related to suicide attempts increased. This increase was more pronounced among women and those aged 25–49 years.

Previous studies also reported that the number of emergency dispatches decreased during the COVID-19 pandemic,^21^ while suicidal ideation^13^^–^^16^ and suicide attempts^18^ potentially increased. The present study extended upon these previous findings by examining a longer observational period.^18^ One potential explanation for the observed decrease in the number of emergency dispatches is decreased incidence of pneumonia, influenza,^22^ or accidents^23^ resulting from reduced transmission risk or social activity. Another reason could be that patients may have refrained from visiting hospitals. By contrast, emergency dispatches related to suicide attempts increased in 2020 compared with the previous 2 years. This increase could be partly explained by economic loss^9^^,^^24^^,^^25^; disrupted social networks^10^; or reduced social capital^26^^–^^37^; and worsening of existing conditions, including psychiatric disorders.

Economic losses caused by the COVID-19 outbreak may have played a major role in the more pronounced increase in suicide attempts among women and individuals aged 25–49 years. Because those aged 25–49 years are of working and child-rearing age, decreased economic activity and school closures brought more hardship compared with other generations. Moreover, women are more often employed as precarious workers^38^ and are generally more vulnerable to loss of employment.^39^^,^^40^ The International Labor Organization reports that employment losses resulting from the COVID-19 pandemic were greater among women than men worldwide.^41^ According to the Labor Force Survey of the Ministry of Internal Affairs and Communications in Japan, the number of unemployed women was increased in October 2020 compared with the same period of 2019.^42^

Overall, the distribution of injury forms associated with suicide attempts did not change in 2020 compared with previous years. However, closer inspection showed increases in the use of instruments, such as knives and sharp objects, among men and in chemicals use among women (Table 3 and Table 4). Given that the number of suicide deaths did not increase (Table 2), this finding may reflect an increase in the number of individuals who self-harm with easily accessible items but do not intend to die. However, further evaluation is needed because information on severity (and deaths) was obtained before or at hospital admission.

One of the strengths of the present study was its longer observation period compared with previous studies. Second, we were able to measure suicide attempts rather than suicidal ideation. Previous studies have shown that a history of self-harm or suicide attempts is the strongest risk factor for suicide.^43^^,^^44^ Several limitations should also be mentioned. First, although we had information on degree of severity, including deaths, this information was obtained before/at hospital admission and was incomplete. Thus, we could not fully evaluate the impact of the COVID-19 outbreak on suicide deaths. Second, in cases of suicide, an ambulance may not be called. Therefore, it is possible that the number of suicide cases was underestimated here. Third, we did not have detailed information on subject attributes, such as medical history or occupation. Fourth, this is a descriptive epidemiological study; therefore, we cannot evaluate the association between the COVID-19 outbreak and increased suicide attempts. Our primary focus was on economic losses; however, other factors, such as decreased social interactions, could influence the increased suicide attempts. In addition, we did not have sufficient data to determine whether the number of patients who had mental illness increased or not in that period. Future studies that measure the changes of individual economic status and social interactions are warranted in an appropriate analytic study design. Fifth, it is important to evaluate the degree of severity (major/minor) within living subjects. We, however, did not evaluate the details of severity because our data on the severity was subjectively measured, and could be considered misclassified. Sixth, the data did not include the suicide attempt since after December 2020, when Japan experienced the largest increase in COVID-19. The study period is not long enough. We need to update the data in our future work. Finally, we included study subjects only from specific areas of Okayama Prefecture. Thus, the generalizability of our findings may be limited. Okayama City is the most urbanized city in Okayama Prefecture, and our findings may be applicable to urbanized cities in other areas.

In conclusion, we evaluated emergency dispatches during a 6-month period of 2018, 2019, and 2020 during the COVID-19 outbreak in Okayama, Japan. We found that emergency dispatches were decreased in 2020 compared with the previous 2 years, while the number and proportion of emergency dispatches related to suicide attempts increased. This increase was more pronounced among women and those aged 25–49 years. These findings could be partly explained by economic losses associated with the outbreak. There is an urgent need for financial, social, and mental support for the working generation, especially women.
